# Stereophotogrammetric approaches to multi-segmental kinematics of the thoracolumbar spine: a systematic review

**DOI:** 10.1186/s12891-022-05925-2

**Published:** 2022-12-12

**Authors:** Jennifer Fayad, Peter Endre Eltes, Aron Lazary, Luca Cristofolini, Rita Stagni

**Affiliations:** 1 Department of Industrial Engineering, Alma Mater Studiorum – Università di Bologna, Bologna, Italy; 2 National Centre for Spinal Disorders, Budapest, Hungary; 3 In Silico Biomechanics Laboratory, National Centre for Spinal Disorders, Budapest, Hungary; 4 Department of Electrical, Electronic and Information Engineering “Guglielmo Marconi”, Alma Mater Studiorum – Università Di Bologna, Bologna, Italy

**Keywords:** Thoracolumbar spine, Stereophotogrammetry, Multi-segment, Motion

## Abstract

**Background:**

Spine disorders are becoming more prevalent in today’s ageing society. Motion abnormalities have been linked to the prevalence and recurrence of these disorders. Various protocols exist to measure thoracolumbar spine motion, but a standard multi-segmental approach is still missing. This study aims to systematically evaluate the literature on stereophotogrammetric motion analysis approaches to quantify thoracolumbar spine kinematics in terms of measurement reliability, suitability of protocols for clinical application and clinical significance of the resulting functional assessment.

**Methods:**

Electronic databases (PubMed, Scopus and ScienceDirect) were searched until February 2022. Studies published in English, investigating the intersegmental kinematics of the thoracolumbar spine using stereophotogrammetric motion analysis were identified. All information relating to measurement reliability; measurement suitability and clinical significance was extracted from the studies identified.

**Results:**

Seventy-four studies met the inclusion criteria. 33% of the studies reported on the repeatability of their measurement. In terms of suitability, only 35% of protocols were deemed suitable for clinical application. The spinous processes of C7, T3, T6, T12, L1, L3 and L5 were the most widely used landmarks. The spine segment definitions were, however, found to be inconsistent among studies. Activities of daily living were the main tasks performed. Comparable results between protocols are however still missing.

**Conclusion:**

The literature to date offers various stereophotogrammetric protocols to quantify the multi-segmental motion of the thoracolumbar spine, without a standard guideline being followed. From a clinical point of view, the approaches are still limited. Further research is needed to define a precise motion analysis protocol in terms of segment definition and clinical relevance.

## Background

Spinal disorders such as low back pain (LBP) and adult spine deformity (ASD) are becoming more prevalent in today’s ageing society [1, 2], with LBP being the leading global cause of years lived with disease [3, 4] and ASD prevalence rates ranging between 32 and 68% in individuals over the age of 60 [5, 6]. Patients could present with mild to severe symptoms [7] impairing their mobility from gait disturbances to limitations in the spine range of motion (ROM) [8, 9]. Treatment for spinal disorders depends on the severity of the disease [1], when non-operative treatments are exhausted, surgical interventions are needed to provide pain relief or correct deformity [1, 10]; however, the failure rates remain high following surgery ranging between 10 and 46% [7] due to instrumentation failure or sagittal imbalance [7, 10]. These disorders include a wide range of clinical and radiographical characteristics [6]. However, current research suggests that movement abnormalities impact the prevalence of spinal disorders and the recurrence of the disease following treatment [11, 12], hence the need for a better understanding of spine kinematics to improve treatment decisions and outcomes [13, 14].

**Fig. 1:**
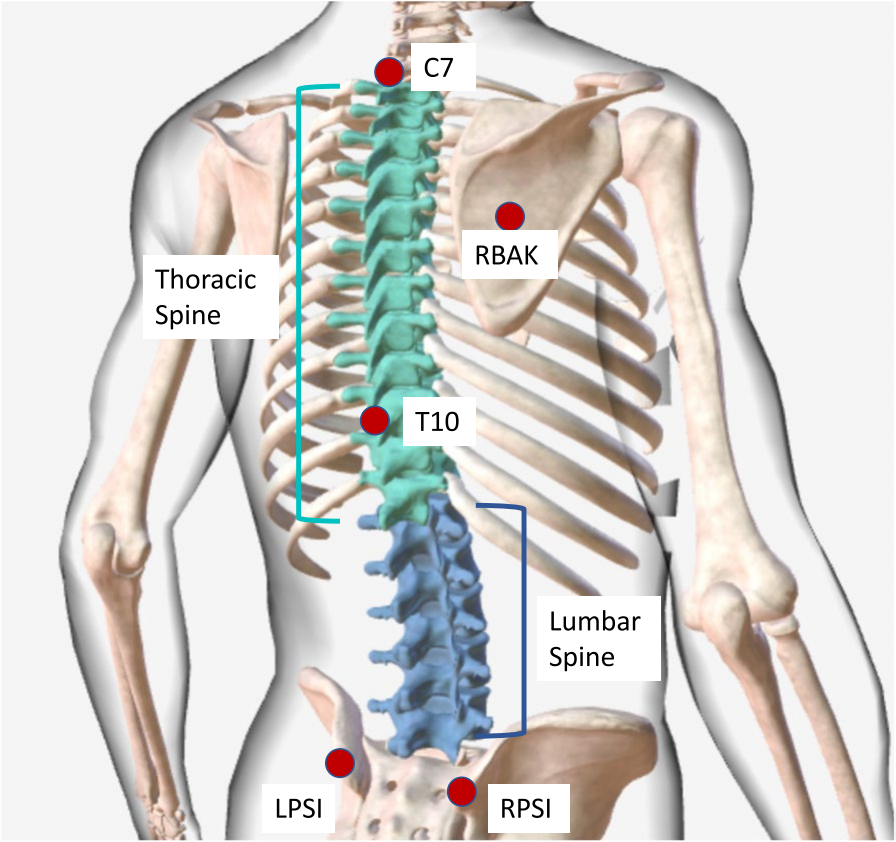
The Plug-in-Gait marker setup (VICON, Oxford, UK) to track motion of the thoracic and lumbar spines using optoelectronic techniques. Red dots show the location of markers on the spine. LPSI: Left Posterior Superior Iliac Spine. RPSI: Right Posterior Superior Iliac Spine. RBAK: Right Back

Different quantification methods are available to quantitatively characterize spine kinematics and posture. i) Spinal alignment angles in the frontal and sagittal planes are quantified in static conditions by means of imaging techniques [15], such as X-rays, CT or MRI scans [9, 15, 16]. These angles are commonly used in clinical practice to support diagnosis, surgical planning, and pre- and post-intervention assessment [9], but do not provide any characterization of spine function in dynamic conditions [16]. Static measurements are also affected by the limited repeatability of the measurements [15] with up to 20% change in lumbar lordosis values in subjects inter-session [17]. Additionally, depending on the spine pathology, imaging techniques are highly affected by lower levels of sensitivity, specificity and an increased rate of false positives with MRI being the most specific and sensitive test for LBP [18]. ii) Intervertebral 3D kinematics can be quantified using video-fluoroscopy [15, 16].This technique is highly accurate, detecting intervertebral ROM with a measurement error varying between 0.32° and 0.52° in the coronal and sagittal plane, respectively [19], but it is not exploited in clinical practice due to the small imaging volume preventing the analysis of spine segments, and due to the critical ionizing radiations exposure [20]. iii) Spine 3D angles can be quantified non-invasively using stereophotogrammetric motion analysis [21] without field of view limitations, allowing also for the assessment during daily living activities [22, 23], but can potentially be affected by significant experimental errors [24]. An overview of stereophotogrammetric motion analysis of the spine can be seen in Fig. 1.

Stereophotogrammetric motion analysis is extensively used for the assessment of body segment kinematics during gait and other functional tasks [25, 26]; although specific protocols and biomechanical models used for the assessment can differ [27]. Body segments (i.e. trunk, pelvis and limb segments) and joint definitions are consistent among protocols [28, 29], while, for spine kinematics, a standard multi-segmental approach is still missing [24, 30].

To assess thoracolumbar spine kinematics in an everyday clinical setting, reliable, clinically significant, and comparable data need to be reported by spinal motion protocols to provide a functional assessment of each spine segment and supply clinicians with a tool to characterize thoracolumbar kinematics changed by different pathologies. To achieve this, a set of standards and guidelines need to be agreed upon with recommendations on the motion tasks to carry out, optimal segment definitions, data to be generated and requirements for a protocol to be suitable for clinical application. Some previous review papers assessing spine motion analysis partially covered the clinical significance of thoracolumbar spine kinematic protocols [24, 31–34] or provided methodological information on the protocols available [24, 25]. However, this review aims to provide a complete assessment of available protocols in terms of 1) reliability of the measurement, 2) suitability of the protocol for clinical application and 3) clinical significance of the reported results to unravel comparable outcomes between the protocols found and ultimately provide recommendations on the standards needed for thoracolumbar motion analysis. As LBP and ASD are pathologies that affect the thoracolumbar spine [1, 35], motion protocols of only the cervical spine were not included in this review.

The review uncovers information relating to the measurement repeatability and accuracy approaches, number of markers used, segment definitions, degrees of freedom assessed, motion analysis system used, task choice, number of participants included, main measurements reported, pathologies assessed, and clinical findings.

## Methods

This systematic review was conducted in accordance with the PRISMA  statement [36].

### Study selection and research criteria

Article search was completed on the 7^th^ of February 2022 on Scopus, PubMed, and Science Direct databases. The research keywords were customised to match each of the databases. Details of the research strings on each of the databases could be seen in Table 1. To understand the effect of LBP and ASD on the motion of the spine studies focusing solely on the cervical spine were excluded.

**Table 1:** Search Strings used per database

Database	Research String
Scopus	TITLE-ABS-KEY (spine OR trunk OR back OR kinematics OR lumbar OR thoracic) AND TITLE-ABS-KEY ("motion analysis" OR "movement analysis") AND ABS (segment*) AND NOT TITLE-ABS (knee OR ankle OR cervical OR head OR inertial OR wireless OR gait)
PubMed	(((("Motion analysis"[Title/Abstract] OR “movement analysis"[Title/Abstract])) AND (spine [Title/Abstract] OR back [Title/Abstract] OR trunk [Title/Abstract]) OR kinematics [Title/Abstract] OR lumbar [Title/Abstract] OR thoracic [Title/Abstract]) AND (segmental [Title/Abstract] OR segment [Title/Abstract])) NOT (cervical [Title/Abstract] OR head [Title/Abstract] OR knee [Title/Abstract] OR ankle [Title/Abstract] OR gait [Title/Abstract] OR inertial [Title/Abstract] OR wireless [Title/Abstract])
Science Direct	(“motion analysis” OR “movement analysis”) AND (spine OR spinal OR back OR trunk OR lumbar OR thoracic) and (segment OR segmental) NOT (Cervical OR head OR ankle OR knee OR gait) NOT (wireless OR inertial)

The outcomes of the searches on the different databases were merged into a single list.

Studies were included in the review if they met the following inclusion criteria:

1. journal papers written in English,
2. assessing the intersegmental motion of the thoracolumbar spine,
3. using stereophotogrammetric motion analysis,

Articles passing inclusion criteria were retained as full-text documents.

### Quality assessment

The quality of the included studies was assessed by one reviewer using a customised quality assessment questionnaire including 19 questions. Questions 1–12 were designed to appraise the general quality of the studies in terms of study design and reproducibility of the method used. Questions 13 to 19 were specifically designed to assess the reliability of the measurement approach, the suitability of the approach to be used in a clinical setting and the clinical significance of the measurement. Quality assessment questions are listed in Table 2.

**Table 2:** Quality Assessment questionnaire used to evaluate the quality of the studies included in the review

Quality Assessment Questionnaire:
1) Are the research objectives clearly stated?
2) Were the eligibility criteria of participants clearly defined?
3) Did the description of the method used, allow for a replication of the measurement?
4) Is the motion analysis system and setup described?
5) Are marker locations clearly described?
6) Were the spine segments chosen clearly stated and defined?
7) Was the population information and anthropometric data provided?
8) Were the movement tasks chosen clearly described?
9) Were the statistical tests used clearly defined?
10) Were the main measurements and their calculations clearly described?
11) Are the main outcomes of the study clearly stated?
12) Were the limitations of the study clearly stated?
13) Was the repeatability of the measurement assessed?
14) Are errors from marker attachment considered?
15) Was the accuracy of the marker setup assessed?
16) Were the marker setups chosen easily applicable in a clinical setting?
17) Was the reason for choosing the motion task justified?
18) Did the participant cohorts include subjects with spine pathology?
19) Were the measurement outcomes clinically relevant?

Each question was scored on a three-level basis: 2 = yes, 1 = limited detail, 0 = no, for an overall score of up to 38 possible points for each article. Bishop et al*.* [37] rating score was used to classify studies by their quality: high quality was associated to articles with a score higher than 80% (31/38), medium quality articles had a score between 51 and 79% (19–30/38) while low quality was associated with a score lower than 50% (18/38).

#### Data extraction

A standardised extraction form was used by one reviewer to identify and report relevant information from each study. The extraction form points are listed in Table 3.

**Table 3:** Standardised form used to extract relevant information from the collected studies

Extraction Form:
1. Repeatability of the measurement
2. Accuracy of marker setup
3. Validation technique used
4. Number of markers used
5. Segments defined
6. Degrees of freedom studied
7. Motion analysis system used
8. Task Choice
9. Number of participants included
10. Pathologies assessed
11. Main measurements reported
12. Clinical findings

The study details extracted could be divided into three categories: reliability of the measurement (points 1 to 3), suitability of the approach to be used in a clinical setting (points 4 to 9) and the clinical significance of the results reported (points 10 to 12). Some of the data extracted were related to more than one category; this was the case for points 4,5,6,8 which related to both the repeatability and suitability of the measurement, points 9 and 10 related to both the suitability of the measurement and the clinical significance of the reported results. Since none of the studies assessed in this review reported on the suitability of the measurement to be used in a clinical setting in terms of time needed to attach the markers and the ease of use of their data processing approach by clinicians, the number of markers used, segments defined, and degrees of freedom studied were reported instead to determine this suitability. Studies using the same protocol as previous ones were grouped into a separate list.

## Results

### Data acquisition and research strategy

**Fig. 2:**
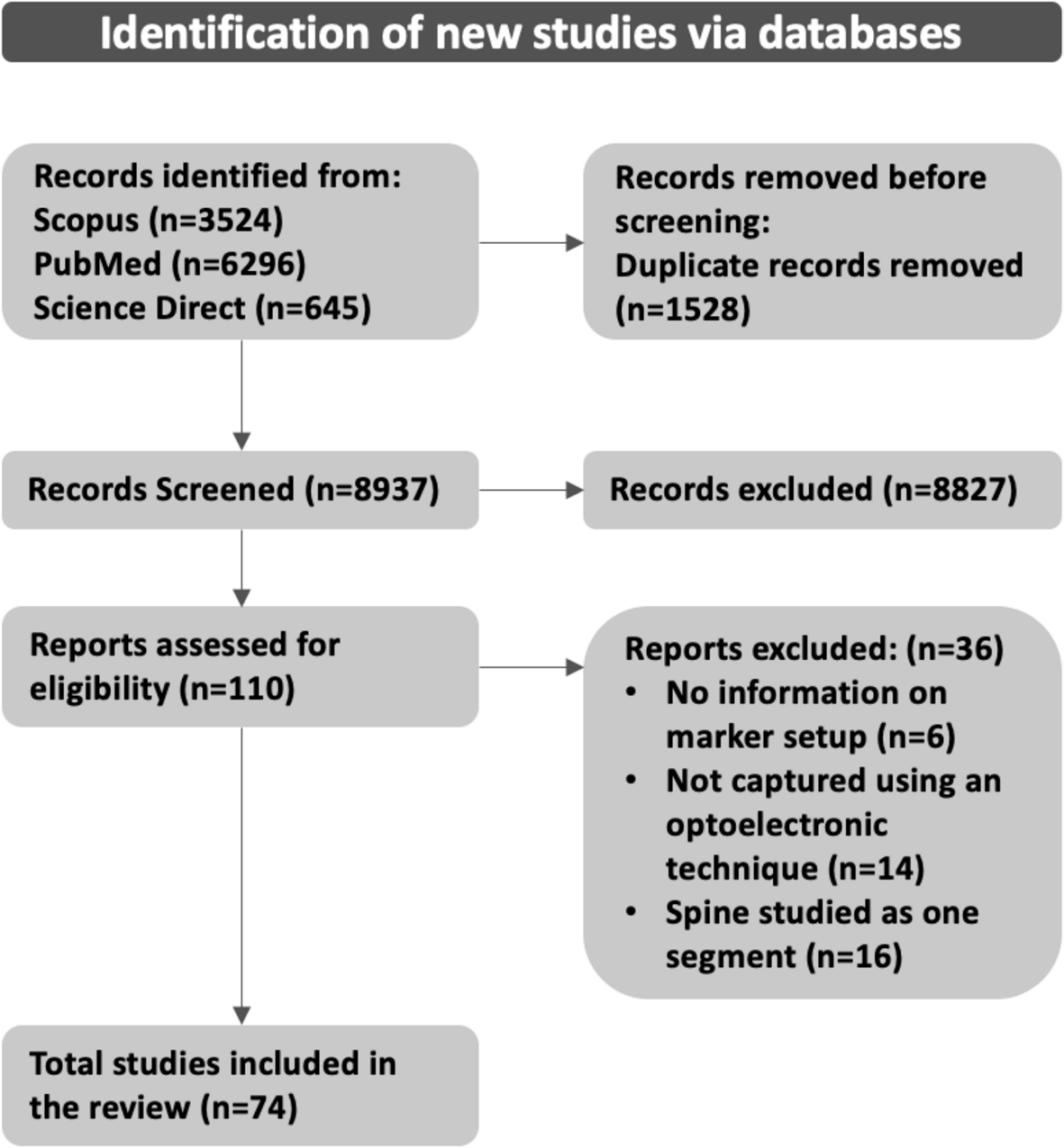
PRISMA flow chart representing the review process

The selection process identified a total of 10,465 records, resulting in 8937 after duplicate removal. After screening titles and abstracts, 8827 studies were excluded as they were deemed irrelevant for the purposes of this review. Inclusion criteria were applied to 110 full-text articles. Seventy-four papers were found to match the inclusion criteria established while 36 studies were excluded as these did not report information on their marker setup, did not use an optoelectronic technique or defined the spine as one moving segment. The process for study selection is shown in the PRISMA flow chart (Fig. 2). Of the 74 articles found, 44 articles proposed new protocols for multi-segments spine motion analysis.

### Quality assessment

**Fig. 3:**
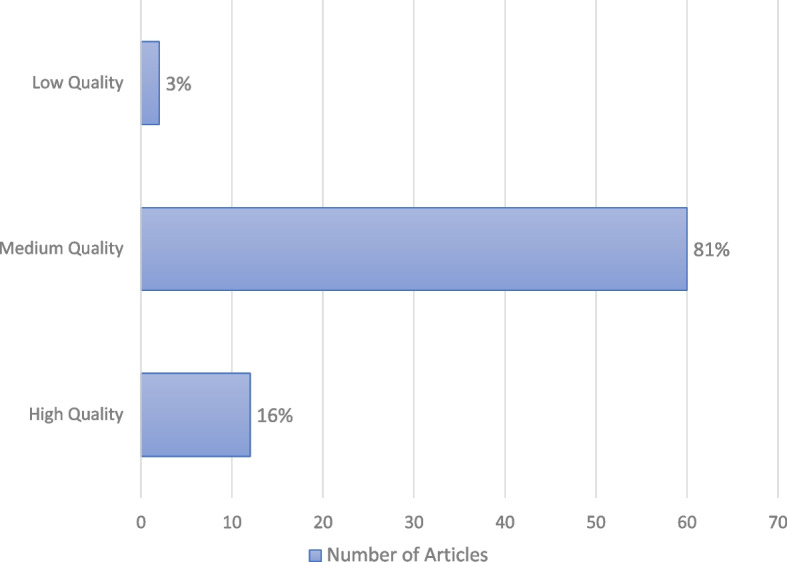
Quality Assessment Score of Articles included in the review; studies were scored as high, medium, or low-quality papers

The 74 articles were assessed using the quality assessment questionnaire. Of those, 12 studies [12–14, 38–46] were found to be of high quality. Sixty studies [23, 26, 30, 47–95] were deemed as medium quality studies and 2 studies [96, 97] had a low-quality score below 50% (Fig. 3).

**Fig. 4:**
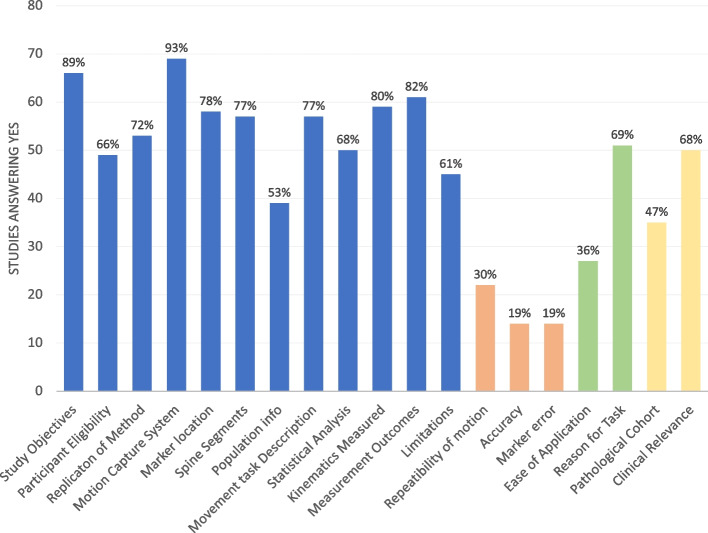
Quality Assessment Questionnaire. The number of articles answering yes to each of the questions. Blue bar plots indicate overall quality questions, orange bar plots indicate reliability related questions, green bar plots indicate suitability related questions and yellow bar plots indicate clinical relevance related questions

The number of articles answering yes to the quality assessment questions could be seen in Fig. 4.

The details and study characteristics obtained from the extraction form of the 74 articles reviewed could be found in Tables 4 and 5.

**Table 4:** Characteristics of included studies as retrieved from the data extraction form

**Study**	**NO. of Markers**	**Spine segments analysed**	**Kinematic variable assessed**	**System used**	**No. of Participants**	**Task performed**
Alemi et al*.,* [98]	35	3 (T1-T5, T5-T9, T9-L1, L1-S1)	Frontal, Sagittal, and transverse plane angles between the segments	10-camera VICON System	7	Flexion–extension, lateral bending, axial rotation
Arshad et al*.*, [67]	47	5 (L1L2, L2L3, L3L4, L4L5, L5S1)	ROM in the sagittal, frontal, and transverse planes	10-camera VICON System, NEXUS	6	Walking trials
Choi et al*.*, [92]	18	5 (Cervical, UT, LT, UL, LL)	ROM in the sagittal, frontal, and transverse planes	6-camera VICON 460 system, VICON NEXUS	6	Walking Trials
Christe et al*.*, [13]	20	4 (UT, LT, UL, LL)	ROM in the sagittal plane	VICON system at 120HZ, MATLAB	21	Sit-to-Stand
Claus et al*.*, [93]	5	3 (T5-T10, T10-L3, L3-S2)	Thoracolumbar angle in the sagittal plane	VICON system at 30 Hz, VICON NEXUS, MATLAB	50	spontaneous sitting position, correct sitting position, typical standing posture
Crosbie et al*.,* [94]	15	4 (UT, LT, lumbar, pelvis)	Frontal, Sagittal, and transverse plane angles between the segments	4-camera Motion Analysis Corporation System	108	Walking trials
Frigo et al*.*, [47]	12	3 (M1-M3, M3-M6, M6-M8	Sagittal and frontal plane angles between the segments	4-camera ELITE system motion analyser at 100 Hz	18	Walking trials
Ghasemi et al*.,* [86]	42	2 (thoracic, lumbar)	ROM and lumbopelvic rhythm in sagittal, frontal, and transverse planes	10-camera VICON system, NEXUS	18	Flexion forward, extension backward, lateral bending, spine rotation, load handling tasks
Gombatto et al*.*, [12]	10	4 (UT, LT, UL, LL)	ROM in the sagittal, frontal, and transverse planes	9-camera VICON system, NEXUS, VISUAL 3D	36	Walking trials
Hemming, [43]	30	4 (UT, LT, UL, LL)	ROM of the Sagittal plane	8-camera VICON 512 at 100 Hz, MATLAB	79	Reach up, sit-to-stand, stand-to-sit, step up, step down, box lift, box replace, bend to retrieve pen from floor
Hidalgo et al*.,* [16]	9	5 (UT, LT, UL, LL, Lumbar)	Frontal, Sagittal, and transverse plane angles between the segments	8-camera BTS System	50	Flexion forward, lateral bending, flexion wit left and right rotation while in a seated position
Holewijn et al*.*, [49]	40	2 (proximal and distal parts of the fused spine)	ROM in the sagittal, transverse, and frontal planes	10-camera VICON system at 100 Hz, NEXUS	12	Walking on a treadmill at a speed of 1.35 m/s
Ignasiak et al*.,* [50]	75	8 (thoracic, lumbar, C7-T3, T3-T5, T5-T7, T7-T9, T9-T11, T11-L1)	ROM in the sagittal plane	12-camera VICON MX system at 100 Hz, NEXUS	42	Full range flexion forward with return to upright posture
Kakar et al*.*, [52]	19	3 (upper trunk, middle trunk, lower trunk)	ROM in the sagittal, transverse, and frontal planes	7-camera VICON M MX System at 240 Hz, NEXUS	20	Running on a treadmill at speeds between 2.2–3.8 m/s
Konz et al*.*, [53]	5 + 8 marker triads	3 (cervical, thoracic, lumbar)	Frontal, sagittal, and transverse angles between segments	8-camera EAGLE digital real-time measurement system	10	Walking at 5 selected speeds
Kuai et al*.*, [54]	8	5 (L1L2, L2L3, L3L4, L4L5, L5S1)	Frontal, sagittal, and transverse angles between segments	NDI OPTOTRAK CERTUS motion analysis system at 100 Hz, MATLAB	33	Walking trials, stair climbing, max flexion
Kudo et al*.,* [99]	70	1 (trunk), 2 (C7-T9, T9-S1), 3 (C7-T6, T6-T12, T12-S1), 6 (C7-T3, T3-T6, T6-T9, T9-T12, T12-S1)	Sagittal, frontal, and transverse plane angles between segments	23-camera Motion Analysis Corporation System at 250 Hz, MATLAB	10	Max flexion, lateral bending and axial rotation posture held for 5 s. Walking trials
Kuwahara et al*.*, [45]	24	2 (thoracic, lumbar)	Sagittal plane angles between segments	16-camera VICON MX system at 100 Hz	20	Walking trials
Leardini et al*.*, [23]	14	5 (C7-T2, T2-MAI, MAI-L1, L1-L3, L3-L5)	Sagittal, frontal, and transverse plane angles between segments	8-camera VICON 612 at 100 Hz, NEXUS, MATLAB	10	Chair rising and sitting, step up and down, walking trials
Lin et al*.,* [55]	39 + 6 marker triads	5 (UT, Middle thoracic, Thoracolumbar, UL, LL)	ROM in the sagittal plane, angular velocity	10-camera VICON MX system, NEXUS, MATLAB	24	Box lifting
List et al*.*, [56]	71	2 (thoracic, lumbar)	Sagittal, frontal, and transverse plane angles between segments	12-camera VICON MX system at 100 Hz, MATLAB	30	Restricted and unrestricted squats
Marich et al*.,* [100]	35	2 (thoracic, lumbar)	Sagittal plane angles between segments	8-camera VICON system at 120 Hz, VISUAL 3D, MATLAB	48	Object pick-up at different heights and distances
Mason et *al.,* [30]	14	2 (thoracic, Lumbar)	ROM in the sagittal, transverse, and frontal planes, angles between segments	12-camera Qualisys Pro-reflex system at 240 Hz, Visual 3D, MATLAB	12	Running at a speed of 5.6 m/s
Needham et al*.*, [26]	3	3 (UT, LT, Lumbar)	Frontal, sagittal, and transverse angles between segments	8-camera VICON system at 100fps, VISUAL 3D	10	Walking trials
Papi et al*.,* [59]	24	4 (UT, LT, UL, LL)	ROM in the sagittal, transverse, and frontal planes	10-camera VICON system at 100 Hz, MATLAB	40	Walking trials, sit-to-stand transitions, lifting a 5kgs box
Patel et al*.*, [61]	20	2 (Thorax, Pelvis)	Frontal Plane angles between segments	7-camera VICON 512	15	Walking trials, rotation of the spine
Peharec et al*.*, [97]	15	4 (UT, LT, UL, LL)	Sagittal and coronal plane angles between segments	9-camera Smart BTS system	63	Flexion/extension, lateral bending from standing
Pesenti et al*.,* [63]	36	2 (Thoracic, Lumbar)	Spine curvatures, CVA, SVA	6-camera VICON system at 100 Hz	62	Walking Trials
Pollock et al*.,* [64]	4	3 (UT, LT, UL)2	Sagittal plane angles between the segments	7-camera VICON at 100 Hz	8	Walking on a treadmill for 60 min
Preuss and Popovic, [65]	24	7 (UT, MUT, MLT, LT, UL, LL, Sacral)	Frontal, sagittal, and transverse angles between segments	6-camera VICON 512 system at 120 Hz	11	Leaning towards targets while seated
Rozumalski et al*.*, [68]	6 marker triads	5 (L1-L2, L2-L3, L3-L4, L4-L5, L5-S1)	ROM of all three anatomical planes	12-camera VICON MX system	10	Maximum voluntary spine ROM, walking trials, jogging, sit-to-stand, lifting
Ryan and Bruno, [69]	14	2 (UL, LL)	Frontal, sagittal, and transverse angles between segments	6-camera VICON T-series system at 100 Hz, NEXUS, VISUAL 3D	17	Walking trials, alternately raise the leg to a height of 20 cm while keeping the knee extended
Saad et al*.,* [70]	18	6 (C2-T1, T1-4, T4-6, T6-8, T8-10, T10-12)	Sagittal and coronal plane angles between segments	10-camera Motion Analysis Corporation system, MATLAB	10	Sit to stand, stand to flexion motions
Schinkel-ivy and Drake, [72]	5 marker triads	5 (C7-T3, T3-T6, T6-T9, T9-T12, T12-L5)	Frontal, sagittal, and transverse angles between segments	VICON MX system, VISUAL 3D, MATLAB	30	Max flexion, max lateral bending, max twist, slumped standing, thoracic flexion, thoracic lateral bend, thoracic twist
Schmid et al*.*, [46]	56	3 (Cervical, thoracic, lumbar)	Thoracic and Lumbar curvature	12-camera MXT20 VICON system at 200-300 Hz, NEXUS, MATLAB	10	Walking trials
Seay et *al.,* [74]	35	2 (Thoraco-lumbar, Lumbo-Sacral)	Frontal, sagittal, and transverse angles between segments, segment moments	8-camera MC240 QUALISYS System at 240 Hz, QTM, Visual 3D	10	Running at a speed of 3.83 m/s
Seerden et al*.,* [75]	45	6 (UT, Middle Thoracic, Thoracolumbar, UL, LL, lumbosacral)	ROM in the sagittal plane	10-camera VICON MX system at 100 Hz, NEXUS, MATLAB	18	Return from forward flexion, box lifting
Severijns et al*.,* [38]	47 + 6 marker triads	2 (Thoracic, Lumbar)	Spine curvatures, SVA	10-camera VICON system	41	Sit-To-Stand
Sung et al*.,* [79]	34	3 (Lumbar, LT, UT)	Spine rotation	EvaRT: Motion analysis corporation, MATLAB	44	Lateral Bending to dominant and non-dominant sides while holding a bar overhead
Sung et al*.,* [80]	44	3 (Lumbar, LT, UT)	Spine Rotation	6-camera Motion analysis corporation, CORYEX software	32	Trunk rotation from left to right while holding a bar
Swain et al*.*, [81]	17	4 (LL, UL, LT, UT)	ROM in frontal and transverse planes	9-camera MX13 + VICON, NEXUS, VISUAL 3D	60	Max trunk rotation, max side bend
Tojima et al*.*, [82]	8	2 (Pelvis, Lumbar)	ROM in the sagittal, transverse, and frontal planes	7-camera VICON, MATLAB	7	Max flexion/extension, Max lateral bending, axial rotation
Wilk et al*.*, [83]	2 marker triads	2 (lumbar, Thoracic)	ROM in the sagittal and coronal planes	8-camera VICON system at 120 Hz	91	Forward, Backward, and lateral bending
Zwambag et al*.*, [84]	21	2 (thoracic, lumbar)	Frontal, sagittal, and transverse angles between segments	Optitrack motion analysis system	4	Full forward flexion, lateral bending, axial rotation to reach a virtual target

**Table 5:** Characteristics of studies extracted from the literature search with marker setups adapted from papers in Table 4

**Study**	**No. of Markers**	**Spine segments analysed**	**Kinematic variable assessed**	**System used**	**Participants**	**Task Performed**	**Adapted From**
Al Eisa et al*.,* [40]	13	3 (UT, LT, Lumbar, Sacral)	ROM in the transverse and frontal planes	5-camera Qualisys Motion analysis System, MATLAB	113	Lateral Flexion and axial rotation	**Crosbie et al.,** [94]
Alijanpour et al., [88]	40	3 (UT, LT, Lumbar)	ROM in sagittal, frontal, and transverse planes, segment coordination	7-camera VICON system at 200 Hz, NEXUS	14	Rowing	**Needham et al**., [26]
Bagheri et al*.,* [101]	13	2 (thoracic, lumbar)	Frontal, sagittal, and transverse angles between segments	6-camera Qualisys System, QTM, MATLAB	30	Walking trials with and without load carrying	**Seay et al.,** [74] **Hidalgo et al.,** [16]
Beaudette et al*.,* [78]	57	2 (thoracic, lumbar)	ROM in sagittal plane	Optitrack motion analysis system	51	Flexion Extension Motion	**Zwambag et al.,** [84]
Breloff et al*.,* [89]	22	7 (UT, MUT, MLT, LT, UL, LL, Sacral)	ROM in sagittal, frontal, and transverse planes	10-camera Motion analysis corporation, MATLAB	10	Seated anterior and lateral bending, level-ground walking	**Preuss and Popovic,** [65]
Christe et al*., * [41]	20	4 (UT, LT, UL, LL)	ROM in the sagittal plane	VICON at 120HZ, MATLAB	22	Walking Trials	**Christe et al.,** [13]
Christe et al*.,* [14]	20	4 (UT, LT, UL, LL)	ROM in sagittal, frontal, and transverse planes	14-camera VICON at 120HZ, MATLAB	21	Step up on boxes of different heights	**Christe et al.,** [13]
Deane et al*.,* [95]	23	3 (UT, LT, Lumbar)	Peak joint angles in the sagittal, transverse, and frontal planes	10-camera VICON system at 100 Hz, NEXUS, MATLAB	10	Walking trials, sit-to-stand transitions	**Papi et al.,** [59]
Gilleard et al*.,* [48]	15	2 (thoracic, lumbar)	ROM in sagittal, frontal, and transverse planes	8-camera camera Motion analysis corporation	9	Walking Trials	**Crosbie et al.,** [94]
Glover et al*.,* [87]	63	3 (upper trunk, middle trunk, lower trunk)	Error profile of spine markers, tracking error of musculoskeletal models	7-camera VICON M MX System at 120 Hz, OpenSim	7	Running Trials	**Kakar et al.,** [52]
Gombatto et al*.,* [42]	10	4 (UT, LT, UL, LL)	Frontal, sagittal, and transverse angles between segments	9-camera VICON system, NEXUS, VISUAL 3D	35	Picking up a small object from the ground	**Gombatto et al.,** [12]
Hagins et al*.,* [90]	31	4 (LL, UL, LT, UT)	ROM in the sagittal, transverse, and frontal planes	8-camera Motion analysis corporation, VISUAL 3D	59	Dance movements	**Swain et al.,** [81]
Hernandez et al., [44]	1 0	4 (UT, LT, UL, LL)	ROM in sagittal, frontal, and transverse planes	9-camera VICON system, NEXUS, VISUAL 3D	36	Step Down	**Gombatto et al*.***, [12]
Hooker et al*.,* [102]	35	2 (thoracic, lumbar)	Lumbar curvature angle	8-camera VICON system at 120 Hz, NEXUS, MATLAB	154	Preferred sitting posture, flexed, and extended sitting	**Gombatto et al*.***, [12] **Marich et al*.,*** [100]
Ignasiak et al*.,* [51]	75	8 (thoracic, lumbar, C7-T3, T3-T5, T5-T7, T7-T9, T9-T11, T11-L1)	Maximum compressive loads on thoracolumbar spine, ROM in sagittal plane	12-camera VICON MX system at 100 Hz, NEXUS	44	Full Flexion Forward, Stand-to-Sit, Sit-to-Stand	**Ignasiak et al*.,*** [51]
Knechtle et al*.,* [103]	58	2 (thoracic, lumbar)	Angular displacement in Sagittal plane, lumbar lordosis curvature	20-camera VICON at 200 Hz, NEXUS, MATLAB	61	Bending forward, sit-to-stand transitions, object pick-up	**Schmid et al*.,*** [73]
Marich et al*.,* [104]	35	2 (thoracic, lumbar)	Angular displacement in Sagittal plane	8-camera VICON system at 120 Hz, NEXUS, MATLAB	48	Forward bending, object pick-up	**Marich et al*.,*** [100]
Muller et al*.,* [57]	24	3 (UT, LT, Lumbar)	ROM in sagittal, frontal, and transverse planes	8-camera VICON system at 200 Hz	10	Treadmill walking trials with perturbations	**Preuss and Popovic,** [65]
Niggli et al*.,* [91]	58	3 (Cervical, thoracic, lumbar)	Thoracic and Lumbar curvature	10-camera VICON at 200 Hz, NEXUS	20	Walking, running, sit-to-stand, object pickup, vertical jump	**Schmid et al*.,*** [73]
Noamani et al*.,* [58]	21	7 (UT, MUT, MLT, LT, UL, LL, Sacral)	ROM in sagittal, frontal, and transverse planes	6-camera Vicon System at 120 Hz	11	Anterior and side Seated Bending	**Preuss and Popovic,** [65]
Papi et *al.,* [60]	24	4 (UT, LT, UL, LL)	ROM in the sagittal, transverse, and frontal planes	VICON system at 100 Hz, NEXUS, MATLAB	40	Walking trials, sit-to-stand transitions, lifting a 5kgs box	**Papi et al*.,*** [59]
Pelegrinelli et al*.,* [62]	18	2 (Thoracic, Lumbar)	ROM in the sagittal, transverse, and frontal planes	10-camera Oqus 400 Qualisys system at 240 Hz, MATLAB	26	Running on a treadmill at 3.3 m/s	**Mason et *al.,*** [30]
Preece et *al.,* [85]	14	2 (Thoracic, Lumbar)	ROM in the sagittal, transverse, and frontal planes	12-camera Qualisys Pro-reflex system at 240 Hz, Visual 3D, MATLAB	15	Running at a speed of 5.6 m/s	**Mason et al.,** [30],
Rouhani et al., 2015 [66]	22	7 (UT, MUT, MLT, LT, UL, LL, Sacral)	ROM in sagittal, frontal, and transverse planes	6-camera Vicon System at 120 Hz	11	Anterior and side Seated Bending	**Preuss and Popovic,** [65]
Sayers et al*.,* [71]	77	2 (thoracic, lumbar)	Spine Curvatures	22-camera VICON MX system at 100 Hz, MATLAB	20	High-bar squat at 2 different heel elevations	**List et al.,** [56]
Schmid et al*.,* [46]	56	3 (Cervical, thoracic, lumbar)	ROM in the sagittal, transverse, and frontal planes	12-camera MXT20 VICON system at 200-300 Hz, NEXUS, MATLAB	29	Walking trials	**Schmid et al.,** [73]
Seerden et al*., * [39]	45	6 (UT, Middle Thoracic, Thoracolumbar, UL, LL, lumbosacral)	ROM in sagittal, frontal, and transverse planes, motion velocity	13-camera VICON MX system at 100 Hz, NEXUS, MATLAB	43	Forward Flexion, Lateral Bending, Spine Rotation	**Seerden et al.,** [75]
Simonet et *al.,* [76]	58	3 (Cervical, thoracic, lumbar)	ROM of the Lumbar Lordosis	10-camera VICON system at 200 Hz, NEXUS, MATLAB	33	Standing for 10 s, Walking, Running at self-selected speeds	**Schmid et al.,** [73]
Stoll et al*.,* [77]	12	3 (UT, LT, Lumbar)	ROM in sagittal, frontal, and transverse planes	8-camera VICON system at 200 Hz	15	Lifting different weights from the ground	**Preuss and Popovic,** [65]
Sugaya et al*.,* [96]	22	7 (UT, MUT, MLT, LT, UL, LL, Sacral)	ROM in sagittal, frontal, and transverse planes, Muscle forces	VICON system at 200 Hz	11	Ipsilateral Rotation	**Preuss and Popovic,** [65]

### Reliability of the motion capture setup

#### Repeatability

Thirty-one studies asked their participants to repeat the motion tasks three times [12–14, 40–44, 47, 54, 55, 58–61, 65–68, 70, 78, 82, 83, 94], 12 studies did 5 repetitions of the motion tasks [26, 39, 46, 53, 69, 75, 76, 80, 88, 89, 95, 97], 7 studies had 10 repetitions [23, 30, 52, 62, 72, 85], 1 study only asked participants to complete the motion task once [86] while 12 studies did not mention the number of task repetitions made [38, 45, 48, 49, 57, 64, 73, 74, 91–93, 96]. Ten studies reported their intra-subject repeatability measures [23, 38, 46, 69, 70, 72, 85, 89, 95, 105], 3 studies measured their inter-rater reliability [38, 82, 95] while 5 studies assessed the repeatability of the findings when measured across different days [26, 30, 38, 82, 91].

#### Accuracy

Fifteen studies evaluated the accuracy of their marker setups [26, 38, 50, 53, 54, 56, 59, 63, 68, 69, 73, 75, 82, 84, 87, 93]. The soft tissue artefact associated with the motion was quantified using imaging techniques in 3 studies [53, 63, 82]. Two studies used electromagnetic sensors along with passive markers to cross-check the values generated by both systems [84, 93]. Two studies compared the kinematic variables collected from participants to those collected from markers placed on custom-built mechanical models of the spine [26, 53]. One study used the *medimouse* apparatus to compute spinal angles and cross-check with the values generated by the motion analysis [50]. Only 1 study inserted wires into the vertebral body to quantify soft tissue artefact (STA) [68].

### Suitability of the approach

#### Marker setup

Most studies used clusters of single markers for their setups, only 7 studies used marker triads to define spine segments [38, 53, 55, 68, 72, 83, 88]. The spinous processes of C7, T3, T6, T12, L1, L3 and L5 were the most widely used. All studies reported marker positioning by palpation of the anatomical landmark surface. Two studies positioned markers following the curvature of the spine, at the points of most thoracic kyphosis and lumbar lordosis [23, 64]. Information on the time needed to position the markers was not reported by any of the studies.

#### Segment definition

The majority of studies [13, 14, 40, 41, 45, 48, 57, 58, 62, 63, 65, 66, 77, 81, 82, 87, 89, 90, 94, 96] used 4 markers to define a spinal segment using 2 markers on the spinous processes and 2 lateral markers midway between these to form a diamond shaped segment. Eleven studies [38, 39, 43, 47, 50, 51, 54, 55, 64, 67, 72, 75, 80, 93, 97] used only 2 markers on the spinal processes to form a segment line. While 12 other studies [12, 23, 26, 42, 44, 49, 53, 56, 70, 71, 79, 83, 88, 92] used 3 markers to define their segments by using 1 marker on the spine and 2 lateral markers to form a triangular shape. It is worth noting that 12 studies used anterior markers on the sternum to define their spinal segments [23, 30, 46, 52, 56, 71, 73, 76, 85–87, 91].

The most common segment definition used was dividing the kinematic model of the spine into 2 distinct segments, either the thoracic and lumbar spines [30, 45, 48, 49, 56, 61–63, 71, 74, 78, 83–86] or the upper lumbar and lower lumbar spines [69, 82].

Another common segment definition was dividing the back into 3 parts and these were: Upper thoracic, Lower Thoracic and Lumbar [26, 40, 52, 57, 64, 77, 79, 80, 87, 88, 94, 95]. Some studies further divided the lumbar spine into upper and lower lumbar, to have a total of 4 segments [12–14, 41–44, 59, 60, 81, 90, 94, 97]. Only 5 papers further defined the cervical spine in addition to the thoracic and lumbar segments and analysed it in their models [46, 73, 76, 91, 92]. Three studies considered each lumbar vertebra as a single segment [54, 67, 68].

#### Data processing

The kinematic data collected from markers was low pass filtered using Butterworth Filters with a cut-off frequency ranging between 2 and 10 Hz depending on the motion capture setup. Most studies defined the pelvis as the local coordinate system for their data analysis [12–14, 23, 26, 30, 38, 41–59, 61–67, 69–75, 77–83, 89, 92–94, 96, 97]. The Grood and Suntay convention was mentioned in 14 studies to calculate intersegmental angles to obtain the flexion/extension, lateral bending, and axial rotations of the defined segments in all 3 anatomical planes [13, 14, 22, 23, 26, 51, 59, 60, 69, 92, 94, 95, 106, 107].

Some studies reported subtracting the static standing trial of participants from the dynamic trials to normalize the angle of motion [13, 14, 41, 60, 76]. None of the studies included in this review commented on the ease of use of their data processing procedure for use in clinical practice.

#### Participant cohorts

Thirty-five studies had only healthy participants in their cohorts [23, 26, 30, 47, 48, 50, 51, 56–58, 64–72, 74, 75, 77, 78, 82, 84, 85, 87, 89, 91–97]. Twenty-seven studies compared pathological participants to healthy participants [12–14, 38–46, 52, 55, 59–61, 76, 79–81, 83, 86, 88, 90] while only 3 studies assessed the motion of only pathological subjects [49, 63, 73]. Details of participant cohorts can be found in Table 6.

**Table 6:** Study participants sample size and characteristics

**Study**	**Subjects**										
	**Total**	**Healthy**					**Pathological**				
		**No**	**Gender**	**Age**	**Height(m)**	**Weight(kg)**	**No**	**Gender**	**Age**	**Height(m)**	**Weight(kg)**
**Al Eisa et al.,** [40]	113	59	25 M 34F	31.1 ± 6.9	-	-	54	27 M 27F	33.4 ± 7.2	-	-
**Alemi et al*.,*** [98]	7	7	3 M 4F	42 ± 14	1.72 ± 0.07	69.6 ± 11.1	-	-	-	-	-
**Alijanpour et al*.,*** [88]	14	6	3 M 3F	25.03 ± 4.5	1.8 ± 0.09	70.83 ± 14.6	8	4 M 4F	24.12 ± 4.9	1.83 ± 0.09	77.87 ± 13.2
**Arshad et al*.,*** [67]	6	6	6 M	24–33	1.8 ± 0.04	75 ± 8.03	-	-	-	-	-
**Bagheri et al*.,*** [101]	30	15	-	-	-	-	15	-	-	-	-
**Beaudette et al*.,*** [78]	51	51	-	24 ± 3.3	1.8 ± 0.07	80.4 ± 11	-	-	-	-	-
**Breloff et al*,.*** [89]	10	10	5 M 5F	26.8 ± 3.8	1.8 ± 0.02	67.7 ± 11.6	-	-	-	-	-
**Choi et al*.*,** [92]	6	6	6 M	23.8 ± 0.4	1.76 ± 0.04	67.8 ± 1.6	-	-	-	-	-
**Christe et al*.*,** [13]	21	11	6 M 5F	38.2 ± 6.7	1.72 ± 0.07	65.6 ± 9.8	10	5 M 5F	36.7 ± 5.4	1.74 ± 0.05	69.5 ± 9.8
**Christe et al*.,*** [41]	21	11	6 M 5F	36.7 ± 5.4	1.74 ± 0.05	69.5 ± 9.8	10	6 M 4F	38.7 ± 7.2	1.74 ± 0.07	67.8 ± 8.9
**Christe et al*.,*** [14]	21	11	6 M 5F	36.7 ± 5.4	1.74 ± 0.05	69.5 ± 9.8	10	6 M 4F	38.7 ± 7.2	1.74 ± 0.07	67.8 ± 8.9
**Claus et al*.*,** [93]	50	50	21 M 29F	22 ± 4 21 ± 3	1.72 ± 0.07 1.64 ± 0.06	66 ± 12 55 ± 8	-	-	-	-	-
**Crosbie et al*.,*** [94]	108	108	50 M 58F	46 ± 18 45 ± 18	1.72 ± 0.08 1.61 ± 0.07	73.7 ± 10.5 59.6 ± 9.8	-	-	-	-	-
**Deane et al*.,*** [95]	10	10	4 M 6F	30.8	-	-	-	-	-	-	-
**Frigo et al*.*,** [47]	18	18	18F	12.3	1.56	49.2	-	-	-	-	-
**Ghasemi et al*.,*** [86]	18	9	9 M	23.6 ± 1.1	1.78 ± 0.057	75.9 ± 7.1	9	9 M	26.9 ± 3.9	1.76 ± 0.04	110.1 ± 10.6
**Gilleard et al*.,*** [48]	9	9	9F	32.6 ± 4.3	1.63 ± 0.06	66.8 ± 10.3	-	-	-	-	-
**Glover et al*.,*** [87]	7	7	4 M 3F	49.9 ± 12.2	1.72 ± 0.11	-	-	-	-	-	-
**Gombatto et al*.*,** [12]	36	18	8 M 10F	27.6 ± 12.4	1.67 ± 0.12	72 ± 14.5	18	7 M 11F	28.1 ± 13.1	1.69 ± 0.11	71.2 ± 15.3
**Gombatto et al*.*,** [42]	35	17	7 M 10F	25.6 ± 8.7	1.67 ± 0.13	71.1 ± 14.4	18	7 M 11F	28.1 ± 13.1	1.69 ± 0.11	71.2 ± 15.3
**Hagins et al*.,*** [90]	59	24	2 M 21F	24.9 ± 6.1	1.66 ± 0.09	62.1 ± 9.7	33	9 M 26F	24.9 ± 6.1	1.66 ± 0.09	62.1 ± 9.7
**Hemming,** [43]	77	28	12 M 16F	38.5 ± 11.2	1.69 ± 0.07	72.9 ± 15.2	23AEP 27FP	4 M/19F 21 M/6F	43.7 ± 11.2 41 ± 10	1.69 ± 0.1 1.75 ± 0.87	68.9 ± 18 82.5 ± 14.6
**Hernandez et al., 2017** [44]	36	18	8 M 10F	26.1 ± 8.6			19	7 M 11F	28.1 ± 13.1	-	-
**Hidalgo et al*.,*** [16]	50	25	10 M 15F	40 ± 11	-	-	25	12 M 13F	42 ± 9	-	-
**Holewijn et al*.*,** [49]	12	-	-	-	-	-	12	12F	15.2 ± 1.7	-	-
**Hooker et al*.,*** [102]	154	-	-	-	-	-	154	59 M 95F	42.6 ± 1.85	-	-
**Ignasiak et al*.,*** [50]	42	21Young 21Elderly	16 M/26F	27 ± 3.97 70.1 ± 3.85	1.73 ± 0.09 1.68 ± 0.08	68.3 ± 13.7 67.4 ± 11.3	-	-	-	-	-
**Ignasiak et al*.,*** [51]	44	23Young 21Elderly	17 M/27F	27.13 ± 3.79 70.1 ± 3.85	1.73 ± 0.09 1.68 ± 0.08	68.3 ± 13.7 67.4 ± 11.3	-	-	-	-	-
**Kakar et al*.*,** [52]	20	10	4 M 6F	20.6 ± 1.5	1.72 ± 0.08	66.4 ± 10.9	10	4 M 6F	17.4 ± 1.3	1.69 ± 0.09	65.5 ± 12.2
**Knechtle et al*.,*** [103]	61	61	31 M 31F	29.5 ± 6.9	-	-	-	-	-	-	-
**Konz et al*.*,** [53]	10	10	-	27 ± 4	1.71 ± 0.06	71.9 ± 12.2	1	1 M	-	-	-
**Kuai et al*.*,** [54]	33	26	-	-	-	-	7	-	-	-	-
**Kudo et al*.,*** [99]	10	10	10 M	22.6 ± 1.5	1.7 ± 0.05	64.6 ± 6	-	-	-	-	-
**Kuwahara et al*.*,** [45]	20	10	6 M 5F	62 ± 19.1	1.62 ± 0.08	60.7 ± 11.7	10	5 M 5F	75.3 ± 3.9	1.58 ± 0.08	63.4 ± 6
**Leardini et al*.*,** [23]	10	10	5 M 5F	24.7 ± 0.8	1.71 ± 0.08	62.4 ± 9.3	-	-	-	-	-
**Lin et al*.,*** [55]	24	15	10 M 5F	48.2 ± 14.46	1.76 ± 0.09	76.3 ± 14.7	9	8 M 1F	53.9 ± 9.3	1.7 ± 0.05	72.6 ± 11.4
**List et al*.*,** [56]	30	30	-	25 ± 4	1.74 ± 0.08	67 ± 11	-	-	-	-	-
**Marich et al*.,*** [100]	48	16	6 M 10F	37.4 ± 11	1.7 ± 0.13	68.6 ± 14.6	16low LBP 16high LBP	6 M 10F 6 M 10F	38.6 ± 13 36.2 ± 11	1.71 ± 0.11 1.71 ± 0.09	68.9 ± 15.1 71.6 ± 9.6
**Marich et al*.,*** [104]	48	16	7 M 9F	32.1 ± 9.4	1.72 ± 0.12	71.8 ± 11.1	32	17 M 15F	33.8 ± 10	1.72 ± 0.1	74.3 ± 15.3
**Mason et *al.,*** [30]	12	12	11 M 1F	23.25 ± 4.3	1.64 ± 0.06	60.45 ± 8.13	-	-	-	-	-
**Muller et al*.,*** [57]	10	10	5 M 5F	29 ± 3	1.79 ± 0.11	74 ± 14	-	-	-	-	-
**Needham et al*.*,** [26]	10	10	-	-	-	-	-	-	-	-	-
**Niggli et al*.,*** [91]	20	20	9 M 11F	31 ± 9	1.73 ± 0.1	69 ± 13	-	-	-	-	-
**Noamani et al*.,*** [58]	11	11	7 M 4F	28.5 ± 3.3	0.75 ± 0.04	69.9 ± 13.7	-	-	-	-	-
**Papi et al*.,*** [59]	40	20	10 M 10F	28 ± 7.6	1.72 ± 0.11	66.2 ± 12	20	16 M 4F	41 ± 10.7	1.68 ± 0.1	74.1 ± 19.5
**Papi et *al.,*** [60]	40	20	10 M 10F	28 ± 7.6	1.72 ± 0.11	66.2 ± 12	20	16 M 4F	41 ± 10.7	1.68 ± 0.1	74.1 ± 19.5
**Patel et al*.*,** [61]	28	13	6 M 7F	16.6	1.62	64	15	5 M 10F	14.3	1.62	58.3
**Peharec et al*.*,** [97]	63	63	40 M 23F	35	-	-	-	-	-	-	-
**Pelegrinelli et al*.,*** [62]	26	13	-	-	-	-	13	-	-	-	-
**Pesenti et al*.,*** [63]	62	-	-	-	-	-	62	8 M 54F	15.5 ± 2.1	-	-
**Pollock et al*.,*** [64]	8	8	8 M	22 ± 3.9	1.72 ± 0.07	76 ± 8.9	-	-	-	-	-
**Preece et *al.,*** [85]	15	15	15 M	25 ± 5	1.78 ± 0.0689	63.1 ± 6.1	-	-	-	-	-
**Preuss et al*.,*** [65]	11	11	7 M 4F	28.5 ± 3.3	-	-	-	-	-	-	-
**Rouhani et al., 2015** [66]	11	11	7 M 4F	28.5 ± 3.3	-	-	-	-	-	-	-
**Rozumalski et al*.*,** [68]	10	10	-	-	-	-	-	-	-	-	-
**Ryan et al*.,*** [69]	17	17	10 M 7F	26.5 ± 5.4	1.68 ± 0.09	67.9 ± 10.5	-	-	-	-	-
**Saad et al*.,*** [70]	10	10	10 M	-	-	-	-	-	-	-	-
**Sayers et al*.,*** [71]	20	10Novice 10Pro	5F/5 M 5F/5 M	26.1 ± 4.9 27.6 ± 3.6	1.73 ± 0.1 1.71 ± 0.09	67.6 ± 12.4 66 ± 10.7	-	-	-	-	-
**Schinkel-ivy et al*.,*** [72]	30	30	15 M 15F	25 ± 3.8 22.8 ± 2.7	1.8 ± 0.05 1.66 ± 0.05	79 ± 8 59 ± 6	-	-	-	-	-
**Schmid et al*.*,** [73]	10	-	-	-	-	-	10	2 M 8F	14.8 ± 1.3	1.65 ± 0.1	55.3 ± 12.7
**Schmid et al*.*,** [46]	29	15	8 M 7F	14.1	1.62	54.2	14	2 M 12F	15.2	1.66	55.6
**Seay et *al.,*** [74]	10	10	-	26.2	1.72 ± 0.14	66.2 ± 10.2	-	-	-	-	-
**Seerden et al*.*,** [75]	18	18	11 M 7F	45.8 ± 14.8	1.76 ± 0.09	74 ± 14.5	-	-	-	-	-
**Seerden et al*.,*** [39]	43	23	16 M 7F	45.2 ± 13.4	1.77 ± 0.09	75.2 ± 14	12 AK 8 Inf	11 M, 1F 7 M, 1F	50.4 ± 11.4 37.6 ± 13.7	1.73 ± 0.06 1.79 ± 0.08	77.3 ± 12.9 87.3 ± 15.9
**Severijns et al*.,*** [38]	41	18	6 M 12F	61.4 ± 10.5	1.65 ± 0.07	63.8 ± 12	23	4 M 19F	61.8 ± 10	1.62 ± 0.07	60.6 ± 9.5
**Simonet et *al.,*** [76]	33	20	9 M 11F	31.4 ± 9.2	1.73 ± 0.09	68.9 ± 12.9	13	8 M 5F	38 ± 11.6	1.74 ± 0.07	67 ± 12
**Stoll et al*.,*** [77]	10	10	6 M 4F	29 ± 3	1.79 ± 0.09	75 ± 14	-	-	-	-	-
**Sugaya et al*.,*** [96]	11	11	11 M	26.5 ± 3.3	1.73 ± 0.04	65.4 ± 3.9	-	-	-	-	-
**Sung et al*.,*** [79]	44	24	18 M 6F	39.7 ± 18.7	-	-	20	12 M 8F	43.1 ± 17.4	-	-
**Sung et al*.,*** [80]	32	18	4 M 12 F	14.22 ± 0.73			14	2 M 12F	14.79 ± 1.05		
**Swain et al*.*,** [81]	60	27	-	-	-	-	33	-	-	-	-
**Tojima et al*.*,** [82]	7	7	7 M	30.3 ± 4.9	1.7 ± 0.05	64.4 ± 6.6	-	-	-	-	-
**Wilk et al*.*,** [83]	91	25	25F	15–28	-	-	66	66F	15–28	-	-
**Zwambag et al*.*,** [84]	4	4	4 M	27 ± 1.7	1.8 ± 0.1	85 ± 10.3	-	-	-	-	-

*M* Male, *F* Female, *AEP* Active extension pattern, *FP* Flexion pattern, *AK* Ankylosed axial spondyloarthropathy, *Inf* Inflamed axial spondyloarthropathy

#### Tasks conducted

The majority of studies [12–14, 23, 26, 41–49, 51, 53–55, 57, 59–61, 63, 64, 67–69, 73, 75–77, 86, 89, 91–95] looked at the motion of the spine segments during activities of daily living (ADL), as these were considered routine and repetitive motions where the spine plays a key role to assure equilibrium and are affected in spine pathology cases. Some of these studies [12, 26, 41, 45–49, 53, 54, 59–61, 63, 64, 67–69, 73, 76, 92, 94, 95] looked at the active role the spine segments play during gait to maintain equilibrium and the compensation mechanism used by patients to achieve it.

When it comes to the studies that recruited patients undergoing spine surgery [45, 49, 52, 53, 61, 83], ADL tasks were used to assess improvement or deterioration of neurological symptoms, changes in motion patterns and the compensation mechanisms involved in the motion.

Twenty-two studies [39, 40, 50, 51, 58, 61, 65, 66, 68, 72, 75, 78–84, 86, 89, 96, 97] included spine range of motion tasks such as forward flexion, lateral bending, or spine rotation. These tasks were implemented to report normal spine segment kinematics, investigate the role of each spine segment in spine motion and assess the reliability of motion capture setups. Eight studies [30, 52, 62, 74, 76, 85, 87, 91] looked at the motion of the spine during running trials while 2 studies [56, 71] assessed spine motion during the squat exercise, 2 studies [81, 90] assessed spine motion while their participants performed dancing tasks while 1 study assessed the motion of the spine during rowing [88].

##### Patient considerations

Studies involving patients and healthy subjects [12–14, 38–46, 52, 55, 59–61, 76, 79–81, 83, 86] had the same tasks for both cohorts. Participants were asked to perform their tasks at their self-selected speed. One study involving patients asked their participants to perform a lifting task only in their most comfortable approach [55].

#### Main measurements

Eighteen studies calculated the angles between the spine segments defined [23, 26, 42, 47, 53, 56, 61, 64, 65, 69, 70, 72, 74, 84, 90, 91, 93–95, 97] while 33 studies [12–14, 30, 39–41, 43, 44, 46, 48–52, 55, 57–60, 62, 66–68, 75, 77, 78, 82, 83, 85, 86, 88, 92, 96] calculated the range of motion of the segments during the tasks conducted.

The ROM of segments during walking tasks ranged from 2.3° to 7.9° in the sagittal plane, 1.8° to 10.8° in the frontal plan while most of the motion was recorded in the transverse plane ranging from 2.6° to 13.5°. Detailed ROMs of the spine segments defined in the studies extracted can be found in Table 7.

**Table 7:** Range of Motion of the spine segments during the various tasks reported in the studies extracted

**Task**	**Study**	**Cohort**	**Spine Segment**	**Plane**	**Angle**°	**SD**°
**Walking**	**Choi et al*.*,** [92]	Control	UT/LT	Sagittal	2.3	1.1
				Frontal	2.8	1.4
				Transverse	2.6	1.8
			LT/UL	Sagittal	3.8	1.5
				Frontal	2.5	0.4
				Transverse	7.9	1.3
			UL/LL	Sagittal	3.6	1.3
				Frontal	5.6	1.4
				Transverse	5.3	1.2
			LL/Pelvis	Sagittal	4.8	1.8
				Frontal	7.9	1.8
				Transverse	5	1.3
	**Christe et al*.,*** [41]	Control	UT/LT	Sagittal	4.45	-
			UL/LL	Sagittal	6.55	-
			LL/Pelvis	Sagittal	7.97	-
		LBP	UT/LT	Sagittal	4.46	-
			UL/LL	Sagittal	4.45	-
			LL/Pelvis	Sagittal	6.55	-
	**Crosbie et al*.,*** [94]	Control	LT	Sagittal	2.5	1.5
				Frontal	7	3
				Transverse	4	2.5
			Lumbar	Sagittal	3.5	2
				Frontal	9	3.5
				Transverse	4.5	2
			Pelvis	Sagittal	3.5	1.5
				Frontal	6	2.5
				Transverse	4	2.5
	**Gombatto et al*.*,** [12]	Control	UL	Sagittal	7.9	-
				Frontal	2.9	-
				Transverse	5.5	-
			LL	Sagittal	4.5	-
				Frontal	1.8	-
				Transverse	2.2	-
		LBP	UL	Sagittal	5.8	-
				Frontal	2.7	-
				Transverse	4.7	-
			LL	Sagittal	4.5	-
				Frontal	2	-
				Transverse	3.7	-
	**Holewijn et al*.*,** [49]	AIS	Proximal Spine	Sagittal	5.5	2.7
				Frontal	8.3	2.9
				Transverse	12.2	4
			Distal Spine	Sagittal	8	0.3
				Frontal	8.2	3.4
				Transverse	13.5	1.7
	**Konz et al*.*,** [53]	Control	Thoracic	Sagittal	5.7	0.9
				Frontal	7.1	2.4
				Transverse	8.7	2.5
			Lumbar	Sagittal	6.8	1.4
				Frontal	10.8	2.4
				Transverse	11.5	1.3
		AIS	Thoracic	Sagittal	7.1	2.4
				Frontal	8.7	2.5
				Transverse	6.8	1.4
			Lumbar	Sagittal	10.8	2.4
				Frontal	11.5	1.3
				Transverse	4.1	0.9
	**Leardini et al*.*,** [23]	Control	Thoracic	Sagittal	4.2	4.7
				Frontal	5.1	2.1
				Transverse	8.3	3.1
	**Muller et al*.,*** [57]	Control	UT	Sagittal	5.8	2.6
				Frontal	3.8	0.9
				Transverse	12.8	2.9
			LT	Sagittal	6.9	1.5
				Frontal	3.8	1.1
				Transverse	12.6	3.3
			Lumbar	Sagittal	6	1.2
				Frontal	3.4	1.1
				Transverse	13.9	3.4
	**Needham et al*.*,** [26]	Control	UT	Sagittal	2.21	0.83
				Frontal	5.6	1.93
				Transverse	11.34	4.68
			LT	Sagittal	3.74	1.74
				Frontal	5.54	2.43
				Transverse	5.5	1.56
			Lumbar	Sagittal	3.22	0.63
				Frontal	6.5	2.11
				Transverse	7.39	2
	**Ryan and Bruno,** [69]	Control	UL	Sagittal	4	0.9
				Frontal	3.15	0.5
				Transverse	8.3	1.4
			LL	Sagittal	5.1	1.1
				Frontal	4.27	0.54
				Transverse	9.9	1.3
	**Schmid et al*.,*** [46]	Control	Thoracic	Sagittal	4	1.6
				Frontal	4.3	1.6
				Transverse	7.3	2.7
			Lumbar	Sagittal	4.3	1.3
				Frontal	5.2	1.9
				Transverse	9.3	3.3
		AIS	Thoracic	Sagittal	4.8	1
				Frontal	3.7	1.4
				Transverse	6.7	1.7
			Lumbar	Sagittal	4.3	0.9
				Frontal	7.1	2.4
				Transverse	10.6	3.6
**Forward Bending**	**Ghasemi et al*.,*** [86]	Control	Trunk	Sagittal	125	-
			Lumbar	Sagittal	45	-
			Pelvis	Sagittal	50	-
		Obese	Trunk	Sagittal	118	-
			Lumbar	Sagittal	45	-
			Pelvis	Sagittal	55	-
	**Marich et al*.,*** [104]	Control	Lumbar	Sagittal	33.8	7.1
		LBP	Lumbar	Sagittal	35.1	9.3
	**Seerden et al*.,*** [39]	Control	UT	Sagittal	12.4	5.4
			MUT	Sagittal	4.7	3
			LT	Sagittal	8.7	3.8
			UL	Sagittal	18.6	6.5
			LL	Sagittal	28	8.6
	**Wilk et al*.*,** [83]	Control	Thoracic	Sagittal	25	10
			Lumbar	Sagittal	63	10
		Fused Spine	Thoracic	Sagittal	18	10
			Lumbar	Sagittal	57	12
**Seated Bending**	**Breloff et al*.,*** [89]	Control	UL/LT	Sagittal	10.53	10.16
			LL/UL	Sagittal	11.93	7.56
			LL/Sacrum	Sagittal	18.31	8.52
	**Hidalgo et al*.,*** [16]	Control	UT	Sagittal	122.4	15.2
			LT	Sagittal	110.4	14.1
			UL	Sagittal	81.9	15.9
			LL	Sagittal	73.1	15.8
		LBP	UT	Sagittal	100.1	22
			LT	Sagittal	85.4	20.4
			UL	Sagittal	60.9	16.8
			LL	Sagittal	53.8	16.3
	**Rouhani et al., 2015** [66]	Control	LT/UL	Sagittal	7.5	-
				Frontal	1.8	-
				Transverse	2.2	-
			UL/LL	Sagittal	15.1	-
				Frontal	1.8	-
				Transverse	2.2	-
**Lateral Bending**	**Ghasemi et al*.,*** [86]	Control	Trunk	Frontal	50	-
			Lumbar	Frontal	88	-
			Pelvis	Frontal	72	-
		Obese	Trunk	Frontal	55	-
			Lumbar	Frontal	95	-
			Pelvis	Frontal	62	-
	**Seerden et al*.,*** [39]	Control	UT	Frontal	6.9	3.1
			MUT	Frontal	7.2	3.5
			LT	Frontal	9.1	4.6
			UL	Frontal	10.1	4.2
			LL	Frontal	10.3	5.3
	**Wilk et al*.*,** [83]	Control	Thoracic	Frontal	56	10
			Lumbar	Frontal	52	10
		Fused Spine	Thoracic	Frontal	32	14
			Lumbar	Frontal	42	8
**Axial Rotation**	**Ghasemi et al*.,*** [86]	Control	Trunk	Transverse	88	-
			Lumbar	Transverse	72	-
			Pelvis	Transverse	30	-
		Obese	Trunk	Transverse	95	-
			Lumbar	Transverse	62	-
			Pelvis	Transverse	30	-
	**Seerden et al*.,*** [39]	Control	UT	Transverse	9.6	6
			MUT	Transverse	11.3	6.5
			LT	Transverse	15.3	10.3
			UL	Transverse	7.4	5.2
			LL	Transverse	7.9	4.3
	**Sugaya et al*.,*** [96]	Control	UT	Transverse	39	8
			MUT	Transverse	35	7
			MLT	Transverse	27	6
			LT	Transverse	18	4
			UL	Transverse	11	3
			LL	Transverse	6	2
**Stand-to-sit**	**Al Eisa et al*.,*** [40]	Control	Thoracic	Frontal	37.1	9
				Transverse	43.4	11.4
			Lumbar	Frontal	17	8.2
				Transverse	32.3	8
	**Hemming,** [43]	Control	UT	Sagittal	22.5	7.8
			LT	Sagittal	20.6	7.4
			UL	Sagittal	10.7	10.9
			LL	Sagittal	9.9	11.2
**Sit-to-Stand**	**Al Eisa et al*.,*** [40]	Control	Thoracic	Frontal	26	7.6
				Transverse	39.9	14.2
			Lumbar	Frontal	20.4	7.9
				Transverse	34.8	9.7
	**Christe et al*.***, [13]	Control	UT/LT	Sagittal	5.7	-
			UL/LL	Sagittal	10.3	-
			LL/Pelvis	Sagittal	21.8	-
		LBP	UT/LT	Sagittal	3.3	-
			UL/LL	Sagittal	5.7	-
			LL/Pelvis	Sagittal	10.3	-
	**Hemming,** [43]	Control	UT	Sagittal	20.6	7.4
			LT	Sagittal	10.7	10.9
			UL	Sagittal	9.9	11.2
			LL	Sagittal	-6.3	7.6
**Object Pickup**	**Marich et al*.,*** [104]	Control	Lumbar	Sagittal	21.3	4.7
		LBP	Lumbar	Sagittal	24.9	7.2
	**Stoll et al*.,*** [77]	Control	UT	Sagittal	103.8	14.7
				Frontal	5	18
				Transverse	56.4	13.77
			LT	Sagittal	110.5	18
				Frontal	23.3	9.4
				Transverse	33.1	10.3
			Lumbar	Sagittal	84.33	15.7
				Frontal	22.5	7.1
				Transverse	30	7.86

Four studies [63, 71, 73, 76] reported the angle of inclination between the segments and as such calculated the angles of lumbar lordosis or thoracic kyphosis.

Of the 24 studies that conducted walking trials [12, 26, 41, 45–49, 53, 54, 59–61, 63, 64, 67–69, 73, 76, 91, 92, 94, 95], only 5 assessed the kinematics of the lower limbs and reported the gait parameters generated.

### Clinical significance

#### Pathologies assessed

The majority of studies involving pathology assessed subjects with LBP or chronic low back pain (CLPB) [12–14, 40–44, 59, 60, 62, 76, 79, 81, 88, 90]. Eight studies had teenagers with adolescent idiopathic scoliosis (AIS) [46, 49, 52, 61, 63, 73, 80, 83]. Two studies assessed ASD [38, 53]. One study assessed ankylosing spondylitis [55], another focused only on lumbar disc herniation subjects [54], 1 study assessed lumbar spinal stenosis patients [45] and one study assessed patients with axial spondyloarthropathy (axSpa) with two patient cohorts in the active inflammation or the bone formation phases [39]. Only one study assessed the changes in spine motion due to obesity [86].

Six studies assessed patients undergoing spine surgery, 5 of them had patients who underwent posterior spinal fusion surgery [49, 52, 53, 61, 83] while 1 study had patients undergoing two different decompression surgery approaches [45].

Four studies assessed patient motion before and after surgery. One study measured patients before and 1 month after [45], another measured patients before, 3 months and 12 months after surgery [49], Patel et al*.* [61] measured patients before and 12 months following surgery while Konz et al*.* [53] analysed their subject before and 6 months after surgery.

#### Clinical findings

The studies including patients assessed the kinematics of the multi-segmental spine to help clinical decisions, provide more information on motion compensation, evaluate treatment, and monitor pathology outcome. Kuwahara et *al.* [45] used the multi-segmental motion approach to compare two decompression surgery techniques and measure the improvement of neurological symptoms following surgery of the two-patient cohort. Hemming et *al.* [43] found evidence to support subgrouping LBP patients to better refine intervention approaches. Christe et *al.* [14] suggested that CLBP patient rehabilitation could benefit from targeting specific motion deficits in functional activities.

Of the 29 studies assessing patients, 3 studies [40, 41, 61] reported motion asymmetry at the levels of the lumbar and thoracic spine between the patient and control cohorts, Christe et *al.* [41] reported a 20% increase in transverse plane asymmetry in CLBP patients (Table 7) while Patel et *al.* [61] reported asymmetric axial plane motion in LBP patients. 4 studies assessed spine rotation abilities depending on the pathology, 3 of these [12, 41, 42] reported up to 15% decrease in segment rotation either after a surgical intervention or due to LBP (Table 7). Only 1 study [79] reported an increase in lumbar and thoracic rotations in patients with LBP. The motion profile of axial spondylopathy patients was seen to be similar to the maladaptive motion profiles of patients with CLBP with a significant decrease in motion velocity when compared to controls [39].

When it comes to the motion of the lumbar spine, 3 studies reported a decrease in lumbar spine flexion in LBP patients [13, 41, 44] (Table 7), while 1 study [42] reported a decrease in lower lumbar flexion but an increase in upper lumbar flexion. One study [60] found an increase in the upper lumbar and lower lumbar ROM during walking, sit-to-stand and running tasks in patients with LBP (Table 7).

Five studies reported the motion coordination present between the spinal segments [55, 59, 62, 80, 88]. Of these, two studies [59, 80] found a lack of coordination between the lumbar and thoracic segments in LBP and AIS patients. One study [62] found that the pattern of coordination between segments is different for LBP patients when compared to the control and 1 study [55] found evidence of coordination between the upper lumbar, thoracic and pelvis to stabilize the trunk in ankylosing spondylitis patients. One study assessing the motion of rowers with CLBP [88] found a lack of coordination between the spinal segments when the intensity of the motion is increased while also finding that the lower spine segments could not work as supports for the upper segments.

Five studies [23, 38, 46, 63, 73] reported the changes in spine curvature during dynamic trials when compared to static posture. These were able to show that curvature angles of the spine could be measured with high accuracy and that regional differences exist depending on the pathology. One study [76] reported a decrease in the lumbar lordosis angle during walking and running in patients with non-specific LBP.Two studies [49, 83] reported the motion of the spine following fusion surgery, one with AIS patients and the other with ASD patients, both did not report any hypermobility in the unfused spinal segment. Only one study [81] found no significant spine kinematic differences between the LBP group and the control group.

## Discussion

Motion abnormalities of the spine impact the onset and recurrence of spinal disorders [11, 12], therefore analysing the kinematics of the thoracolumbar spine gives an insight into the causes of these disorders and aids in the choice of treatment [13, 14]. Stereophotogrammetric motion analysis could objectively quantify this motion [22, 23], however numerous methods and protocols are found in the literature. The current review aims to evaluate these studies and assess their reliability, suitability in a clinical setting and clinical significance. Seventy-four articles were identified focusing on the multi-segmental motion of the thoracolumbar spine of which 44 articles proposed a different protocol to quantify this motion. These protocols differed in the number of markers used, segments defined, participant cohorts recruited, disorders analysed, kinematic variables assessed, and outcome measurements reported highlighting the need for a set of standard principles to provide reliable and reproducible kinematic information on various motions, spine segments and spine disorders.

### Reliability of the motion capture setup

The reliability of the identified studies was first evaluated. It was defined as the repeatability and accuracy of the measurement approach in addition to the analysis of sources of error. Most studies did not report on these three reliability aspects scoring 32%, 18% and 18% respectively in the quality assessment questionnaire. To quantify the repeatability of the measurement, we first looked at task repetitions; most studies asked their participants to repeat the motion at least three times [12–14, 23, 26, 30, 39–44, 46, 47, 52–55, 58–62, 65–70, 72, 75, 76, 78, 80, 82, 83, 85, 89, 94, 95, 97] and ultimately based their measurements on the average of trials, however, the repetitions of tasks were unrelated to the number of participants in the cohorts. Some of the studies succeeded in analysing the repeatability of the outcome measurement either by studying the intra-subject variability [23, 38, 46, 69, 70, 72, 85, 89, 95, 105], the inter-rater reliability [38, 82, 95] or the repeatability of the findings when measured across different days [26, 30, 38, 82, 91]. When it comes to the accuracy of the optoelectronic protocol, 11 studies [26, 46, 53, 54, 63, 67, 68, 71, 73, 82, 84] compared their results to more conventional imaging techniques to show the accuracy of the marker setup and their reported outcomes; however, the remaining studies did not report on these differences as they were investigating the changes in spine motion between cohorts and were not reporting the absolute angle of motion of spinal segments [12–14, 38, 40–46, 55, 59, 61, 79–81, 83]. Deane et *al.* [95] was the only study to quantify the standard error associated with spine motion ranging between 0.8° and 5.5° for gait and between 1° and 12.6° for sit-to-stand motion compared to imaging techniques that account for a < 1° of error during static measurements [19]. Only 9 studies [38, 40, 50, 53, 66, 73, 84, 87, 94] reported the marker placement error associated with the setup, this is especially important as the spine region is greatly affected by STA [72] and hence would be expected to be more thoroughly reported. None of the studies identified in this review however was found to report on all three reliability aspects assessed in the quality questionnaire. These shortcomings affect the reliability of the protocols suggested and make implementing a standard protocol in a clinical setting even more difficult.

### Suitability of the approach

When assessing the suitability of the protocols suggested for a clinical setting, we looked at marker configurations, segment definitions, participant cohorts, tasks conducted outcome measures and the ease of use of the methodology in a clinical setting. Major differences in marker setups were seen across studies, with different numbers of markers on the thoracolumbar spine and their location on the anatomical landmarks; the most common anatomical landmarks to attach the markers on were C7/T1, T6/T7, T12/L1, L3, L5. Only 11 studies [23, 30, 46, 52, 56, 71, 73, 76, 85–87] reported positioning of markers anteriorly to the spine on the sternum to decrease the effect of STA on the measurement while the majority of the studies positioned markers laterally to the spine [13, 14, 40, 41, 45, 48, 57, 58, 62, 63, 65, 66, 77, 81, 82, 88, 89, 94, 96]. Some of these marker setups were seen to be very complex for a clinical setting and are more suited for research purposes as they require more time to position due to the high number of marker [38, 46, 50, 51, 55, 56, 67, 68, 73, 79, 80, 88, 90, 91], other studies [12–14, 23, 39, 41–44, 57–59, 64–66, 75, 81, 92, 94, 97] were seen to be successful in limiting the number of markers on the spine or by using 3D clusters. This limitation was mentioned by Glover et al*.,* [87], who saw that a higher number of markers on the spine decreases marker tracking error but the implementation of the protocol and processing the data would take a significantly longer time. A poor consistency was found in terms of thoracolumbar segment definition. These changed depending on the study cohorts and tasks of interest. The majority of studies tried to define at least the thoracic and lumbar spines [30, 45, 48, 49, 56, 61–63, 71, 74, 78, 83–86], the lumbar spine was further divided into upper and lower segments [12–14, 41–44, 59, 60, 81, 90, 94, 97] especially when investigating patients with LBP due to the changes in motion seen at each level [12, 42, 43, 59, 60, 90]. The thoracic spine was also divided into upper and lower segments to have a better understanding of the less studied kinematics of the thoracic spine and help in the investigation of kyphotic and scoliotic spines [13, 14, 23, 41, 50, 56, 81, 90]. When it comes to participant considerations, most of the studies tried to match the age of participants with only 9 studies including participants with an age range difference exceeding 10 years [38, 39, 43, 45, 47, 55, 75, 79, 94]. More than half of the studies investigated ADLs such as lifting, sit-to-stand, stair climbing and walking. These tasks had been seen to present a challenge to spine pathology patients and could highlight the differences in segment ROM and coordination when compared to controls [13, 14, 23, 31, 41]. The angle between the defined segment was the main outcome reported by studies independent of the tasks conducted, marker setup used, or segments defined. Despite ROM being a straightforward indication of motion ability and is easily estimated even in a clinical setting, it can limit our understanding of motion contribution, compensation mechanisms and coordination between the spine segments [31]. The coordination between the segments wasn’t as widely reported although evidence has been found to show changes in coordination due to age and spine pathology [55, 59, 62, 80, 88]. None of the studies in this review reported on the time needed for each measurement or the ease of use of their processing approach in a clinical setting. Hence after assessing the suitability of the studies found, ADLs remain the most useful tasks to understand spine motion and its pathologies [13, 14, 23, 31, 41] while more investigations are needed to agree on marker setups and segments definitions to be used in a clinical setting in addition to what to report when it comes to outcome measurements.

### Clinical significance

When it comes to the clinical significance of the studies evaluated, this was defined as the relevancy of the study and its outcome measurements to a certain spine pathology. Different kinematic findings were reported by the studies depending on the spine pathology, the multi-segmental approach and the marker setup used. When considered as one moving segment, the lumbar spine flexion was seen to decrease overall in subjects with LBP [13, 41, 44]; however, when further dividing the lumbar spine into upper and lower segments, motion contribution by the UL was seen to be greater than the LL segment [12, 42]. As such, segment definition plays a key role in understanding the effects of pathology on spine motion; it is advised to divide the spine into more than 2 segments to be able to describe the motion of the whole spine and understand the contribution of each segment [46]. Besides, grouping patients into subcategories depending on their motion impairments [43] or surgical treatments [45] could reveal the similarities in the kinematic findings against healthy controls [31]. Considerations for the changes in spine motion due to age were limited. Only 2 studies reported the differences in spine ROM between older and younger adults [50, 51] while the majority of the studies reported the motion of the spine in healthy younger adults under the age of 35 [23, 47, 48, 53, 56–58, 65–67, 71, 72, 77, 78, 82, 89, 93, 95–97]. Significant age-related lumbar segment reductions in motions have however been reported in the literature [32], it is therefore advised to investigate the spine motion of both older and younger adults and spine pathology subjects to define the motions of each segment of the spine and the coordination between the segments. When looking at the differences between patients and controls, distinct motion perturbations were found in the axial and sagittal plane during spine motion in addition to transverse plane asymmetry in subjects with spine pathologies [12–14, 39–42, 44, 79, 80, 88]. Hence it is advised to investigate the motion of the defined segments in the three dimensions of motion in addition to studying the coordination between spine segments and to the pelvis to ultimately define distinct motion characteristics for LBP, AIS, ASD or spine surgery patients. The use of a multi-segmental spine motion protocol was seen to be successful in a clinical setting to accurately assess spine curvature [23, 38, 46, 63, 73] and the effect of surgical treatment on the patient motion [45, 49, 83]. Evidence has been found in this review to support the use of a multi-segmental approach for spine motion analysis to help clinicians in the diagnosis and treatment of spine disorders.

### Limitations

The present study has a few limitations. Only three research databases were queried for articles published in a peer-reviewed journal and only in English. Hence, a publication or language bias might have occurred. The quality assessment questionnaire developed was not assessed for objective reliability and validity although it was constructed using prior assessments found in the literature [31, 37]. A reliability assessment of the extracted data and the quality assessment questionnaire was not carried out as they were completed by only one reviewer. The review only included studies using passive markers and an optoelectronic system. Studies assessing multi-segmental spine motion using inertial markers or wearable technology were not assessed due to the high signal to nose ratio linked to these sensors especially when attached to a vertebral landmark [65]. Additionally, studies assessing only the motion of the cervical spine were not included as only the motion of the thoracolumbar spine was found to be relevant to the onset of LBP and ASD [1, 35].

## Conclusions

The current review showed a shortage in standard protocols to assess spine motion using optoelectronic techniques to identify and support clinical investigations. The findings mentioned in the review could be used when trying to choose the most fitting protocol to assess the motion of the thoracic and lumbar spines. Based on the studies assessed in the review, separating each of the thoracic and lumbar segments into upper and lower parts is essential to accurately describe the motion of the spine. Markers attached to C7/T1, T6/T7, T12/L1, L3, L5 in addition to anterior markers on the sternum are needed to describe this motion. This limited number of markers would allow for easier application in a clinical setting. In terms of instrumentation, a motion analysis system made up of at least 6 cameras is needed. However, no study in this review mentioned the cost incurred by such an analysis and a cost effectiveness study would need to be completed to assess the feasibility of using spine motion analysis in a clinical setting. Additionally standardizing the marker setups, segment definitions and tasks conducted as part of a multicentric study could prove to help identify more accurate clinical applications for spine motion analysis.

## Data Availability

There is no specific data to share, however, datasets supporting the conclusions of this review article could all be found in the reference list included within the article.

## Abbreviations

LBP: Low Back Pain
ASD: Adult Spine Deformity
ROM: Range of Motion
CT: Computer tomography
MRI: Magnetic Resonance Imaging
LPSI: Left Posterior Superior Iliac Spine
RPSI: Right Posterior Superior Iliac Spine
RBAK: Right Back
UT: Upper Thoracic
LT: Lower Thoracic
UL: Upper Lumbar
LL: Lower Lumbar
MUT: Middle Upper Thoracic
MLT: Middle Lower Thoracic
STA: Soft Tissue Artefact
M: Male
F: Female
AEP: Active extension pattern
FP: Flexion pattern
AK: Ankylosed axial spondyloarthropathy
Inf: Inflamed axial spondyloarthropathy
ADL: Activities of Daily Living
CLPB: Chronic Low Back Pain
AIS: Adolescent Idiopathic Scoliosis
